# Genome-wide loss of heterozygosity and copy number alteration in esophageal squamous cell carcinoma using the Affymetrix GeneChip Mapping 10 K array

**DOI:** 10.1186/1471-2164-7-299

**Published:** 2006-11-29

**Authors:** Nan Hu, Chaoyu Wang, Ying Hu, Howard H Yang, Li-Hui Kong, Ning Lu, Hua Su, Quan-Hong Wang, Alisa M Goldstein, Kenneth H Buetow, Michael R Emmert-Buck, Philip R Taylor, Maxwell P Lee

**Affiliations:** 1 Genetic Epidemiology Branch, Division of Cancer Epidemiology and Genetics, NCI, USA; 2 Laboratory of Population Genetics, Center for Cancer Research, NCI, USA; 3 Shanxi Cancer Hospital, Taiyuan, Shanxi 030013, PR China; 4 Cancer Institute & Hospital, Chinese Academy of Medical Sciences, Beijing, 100021, PR China; 5 Laboratory of Pathology, Center for Cancer Research, NCI, USA

## Abstract

**Background:**

Esophageal squamous cell carcinoma (ESCC) is a common malignancy worldwide. Comprehensive genomic characterization of ESCC will further our understanding of the carcinogenesis process in this disease.

**Results:**

Genome-wide detection of chromosomal changes was performed using the Affymetrix GeneChip 10 K single nucleotide polymorphism (SNP) array, including loss of heterozygosity (LOH) and copy number alterations (CNA), for 26 pairs of matched germ-line and micro-dissected tumor DNA samples. LOH regions were identified by two methods – using Affymetrix's genotype call software and using Affymetrix's copy number alteration tool (CNAT) software – and both approaches yielded similar results. Non-random LOH regions were found on 10 chromosomal arms (in decreasing order of frequency: 17p, 9p, 9q, 13q, 17q, 4q, 4p, 3p, 15q, and 5q), including 20 novel LOH regions (10 kb to 4.26 Mb). Fifteen CNA-loss regions (200 kb to 4.3 Mb) and 36 CNA-gain regions (200 kb to 9.3 Mb) were also identified.

**Conclusion:**

These studies demonstrate that the Affymetrix 10 K SNP chip is a valid platform to integrate analyses of LOH and CNA. The comprehensive knowledge gained from this analysis will enable improved strategies to prevent, diagnose, and treat ESCC.

## Background

Genetic instabilities are characteristic of most human cancers. Genome-wide detection of chromosomal changes, including loss of heterozygosity (LOH) and copy number alterations (CNA), either gain or loss, are the focus of substantial attention in cancer research. LOH is frequently observed in a variety of human cancers, and regions with frequent LOH may contain tumor suppressor genes. In addition, LOH may associate with the regions affected by haplo-insufficiency of a group of genes. Thus, detection of LOH will likely remain a cornerstone for predicting tumor aggressiveness for many human tumors [1]. Recently, the discovery of large-scale genome-wide copy number variation has stimulated interest in elucidating the role of CNA in the development of malignancy. The 10 K single nucleotide polymorphism (SNP) array (GeneChip Mapping 10 K array, Affymetrix) offers a high-resolution genomic approach to screen chromosomal alterations systematically. Several studies on allelic imbalance or loss in cancers and cancer cell lines using the 10 K SNP array have been published [2-12].

Esophageal squamous cell carcinoma (ESCC) is a common malignancy worldwide and one of the most common malignancies in the Chinese population. There is great geographic variation in the occurrence of this tumor in China, including exceptionally high-risk areas such as Shanxi Province in north central China where some of the highest esophageal cancer rates in the world occur. The standardized incidence rate for esophageal cancer in Shanxi Province is above 100/100,000 person-years, although it appears that both incidence and mortality rates have begun to decline in the past 10 years [13,14]. Within the high-risk regions in China, there is a strong tendency toward familial aggregation, suggesting that genetic susceptibility, in conjunction with environmental exposures, plays a role in the etiology of ESCC. In the past several years, we have tried to identify susceptibility genes and biomarkers that can be used to screen high-risk populations in north central China for ESCC [15-22]. A previous study examined 366 microsatellite markers in a 10 cM density genome-wide scan in 11 ESCC patients, and identified 14 chromosome arms with high-frequency LOH [15]. However, we were unable to further narrow these LOH regions using microsatellite markers due to their low density. Higher density markers are necessary for positional cloning of tumor suppressor genes in LOH regions.

In the present study we established a high-resolution chromosomal instability profile for ESCC by examining germ-line DNA and matched micro-dissected tumor DNA with a 10 K SNP array to determine both LOH and CNA. We also evaluated whether a pool of normal control samples could be used as the normal referent in an LOH study with the 10 K SNP chip instead of matched germ-line DNA.

## Results and discussion

### LOH by patient and chromosomal arms

In the present study, 26 ESCC patients with blood-derived germ-line DNA and matched micro-dissected tumor DNA were investigated using 10 K SNP arrays. The characteristics of these patients are shown in Table 1. The average signal detection rate was higher in germ-line DNA (99%) than that in micro-dissected tumor DNA (79%). Based on NCBI Build 35.1, we summarized characteristics of 11,555 SNPs and mapped them to chromosomes and genes. We first generated a genotyping profile for each patient based on a comparison of the germ-line DNA genotypes to those from the matched micro-dissected tumor DNA. The patients' LOH frequencies, shown in Table 1, ranged from 19% to 95%, and averaged 29%. LOH in four cases (SHE0832, SHE0864, SHE1264, and SHE1490) was performed using DNA from micro-dissected adjacent normal tissue in addition to blood-derived germ-line DNA to see if this affected results, but findings were very similar with both of these two sources of DNA (Table 1).

The frequencies of LOH on each chromosomal arm are shown in Table 2. Non-random LOH was observed on 10 chromosomal arms, including 17p (76%), 9p (72%), 9q (72%), 13q (68%), 17q (66%), 4q (65%), 4p (60%), 3p (58%), 15q (57%), and 5q (52%). Our previous microsatellite marker-based genome-wide LOH scan in 11 ESCC patients with a positive family history of upper gastrointestinal cancer produced overall LOH frequencies that were somewhat higher than the patients evaluated in the present study [15]. We can not explain this between-study LOH frequency variation, but there are several noteworthy differences between the studies that likely influenced LOH rates, including: (i) heterozygosity is higher for the microsatellite compared to the SNP markers examined (~75% versus ~30%); (ii) the total number of markers was much higher in the SNP study than the microsatellite study (11,000 versus 366); and (iii) over twice as many cases were examined in the SNP study (26 versus 11). Some results between studies differed (eg, LOH was ≥ 50% on chromosome 15q in the SNP but not the microsatellite study; LOH was ≥ 50% on 8p, 8q, 11p, 11q and 18p in the microsatellite but not the SNP study). Despite differences in study size, approach, and in some of the results, consistently high LOH frequencies were reported for nine chromosomal arms in both studies (ie, 3p, 4p, 4q, 5q, 9p, 9q, 13q, 17p, 17q). Both studies taken together indicate that LOH on these nine chromosomal arms are the major events associated with genome-wide instability in ESCC in this high-risk Chinese population. These areas are rich in known tumor suppressor genes and oncogenes, including *VHL *on 3p; *NPCA1 *on 4p; *KIT, GIST*, and *PDGFRA *on 4q; *APC *and *MCC *on 5q; *CDKN2A *and *CDKN2B *on 9p; *BRCA2 *and *Rb1 *on 13q; *TP53 *on 17p; and *BRCA1, TOC*, and *NF1 *on 17q.

### LOH regions

When we used the conservative, traditional approach to LOH in LOH/Model A, we detected 20 LOH regions encompassing a total of 125 SNPs. As shown in Table 3, these 20 LOH regions are located on eight chromosome arms – 13q (four regions), 3p (two regions), 4q (three regions), 9p (two regions), 9q (three regions), 17p (three regions), 17q (two regions), and 4p (one region). The size of these LOH regions ranged from 10 kb to 4.26 Mb (average 1.44 Mb); genes involved in these deletion regions are shown in Table 3. Among the 125 SNPs in these 20 LOH regions, 46 are located in genes (one in a coding exon, 39 in introns, and six in 3'- or 5'-UTRs), and 79 are located in regions flanking genes (ie, within 1 kb). One SNP (rs781852) is located in the coding region of gene *ZZEF1 *(Zinc finger, ZZ-type with EF-hand domain 1) on chromosome 17p13.2. Allele A for this SNP encodes an amino acid proline (Pro) and the allele B encodes amino acid leucine (Leu). Eight of 10 heterozygous cases (Pro/Leu) showed LOH (80%), including five cases that lost allele B and three cases that lost allele A. The 46 SNPs that are located within genes map to 32 genes and include four SNPs in the introns of *ZNF618*, and two SNPs each in the introns of *ITPR1, FLJ14834, LHFP, ITGAE, MYH3 *and *MYOCD*. Some of these 20 deletion regions have been previously identified by our lab and others [17,22]. However, the current study provides far greater precision in locating LOH regions (10 kb-4.26 Mb as opposed to 10 cM, which corresponds to 5–10 Mb). As expected, using a less conservative definition for LOH, LOH/Model B detected more regions (and SNPs) than our approach in LOH/Model A – 72 LOH regions containing 2,916 SNPs. The distribution of deletion regions and details from this model are shown in Table 4 and Additional Table 1 (in additional file 1).

Our cLOH data in cLOH/Model A identified only three significant cLOH regions. These included one on 13q12-q13 and two on 13q13, and encompassed a total of 30 SNPs. The sizes of these cLOH regions are 1.9 Mb, 0.4 Mb, and 0.2 Mb, respectively (average 0.83 Mb) (Table 5). The less conservative cLOH/Model B highlighted 64 cLOH regions with 2,128 SNPs; details are shown in Table 6 and Additional Table 2 (in additional file 1).

**Figure 1:**
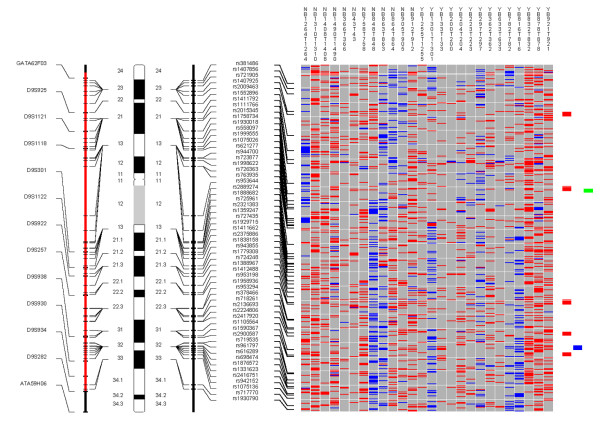
Chromosome 9. Each column in the picture represents an individual case and shows genotyping in germ-line DNA and matched micro-dissected tumor; LOH is shown in red, retention in blue, and homozygous or "no call" in grey. B indicates blood DNA and T indicates tumor DNA (from matched, micro-dissected sample). To the left of the picture, columns show (from left to right): microsatellite markers, cartoon of the chromosome, and SNPs examined in the 10 K SNP chip. To the right of the picture, red bars show deletion regions (as defined from our conservative "LOH/Model A"), blue bars show regions with CNA losses (from CNAT), and green bars show regions with CNA gains (from CNAT).

**Figure 2:**
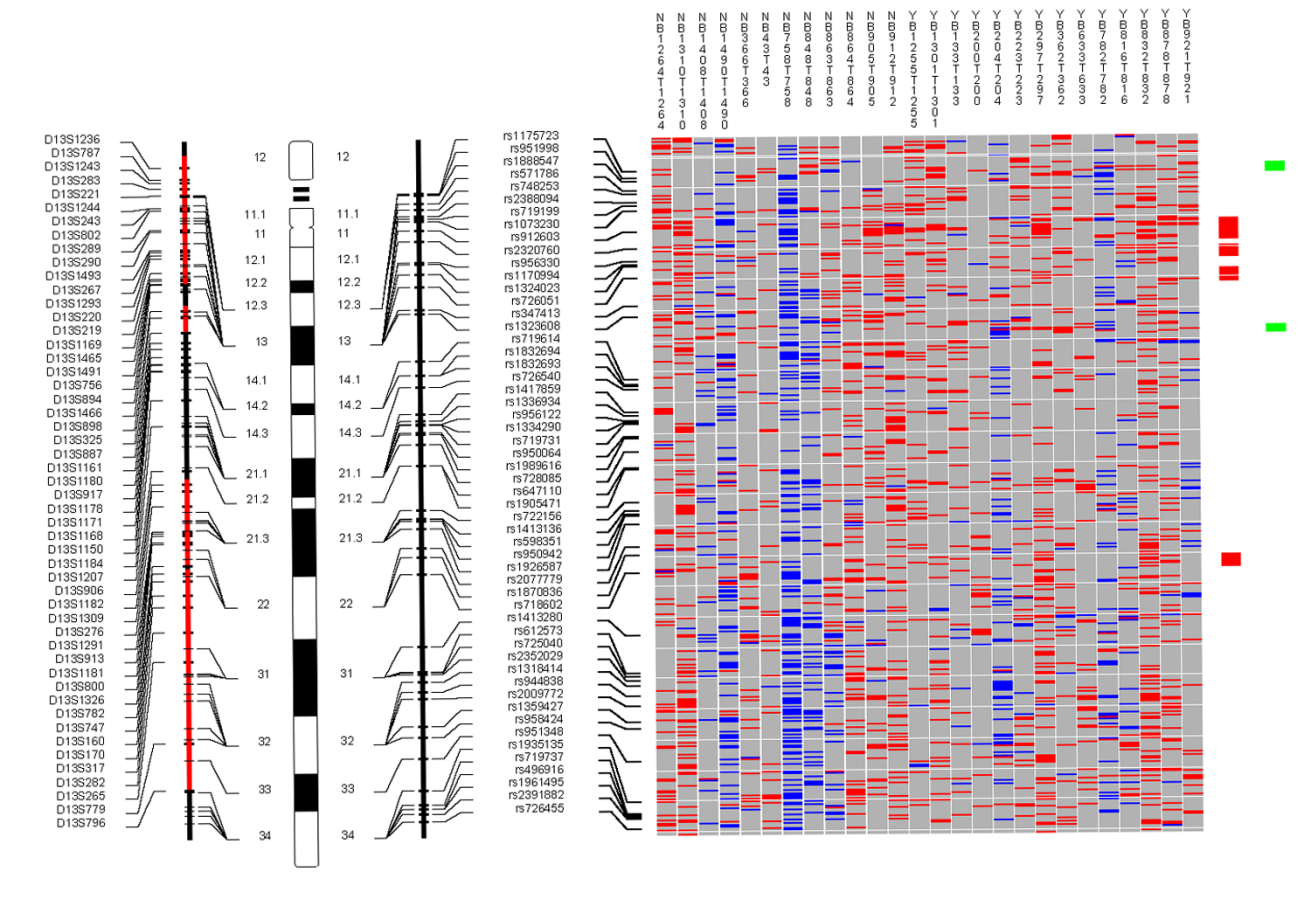
Chromosome 13. Each column in the picture represents an individual case and shows genotyping in germ-line DNA and matched micro-dissected tumor; LOH is shown in red, retention in blue, and homozygous or "no call" in grey. B indicates blood DNA and T indicates tumor DNA (from matched, micro-dissected sample). To the left of the picture, columns show (from left to right): microsatellite markers, cartoon of the chromosome, and SNPs examined in the 10 K SNP chip. To the right of the picture, red bars show deletion regions (as defined from our conservative "LOH/Model A"), blue bars show regions with CNA losses (from CNAT), and green bars show regions with CNA gains (from CNAT).

**Figure 3:**
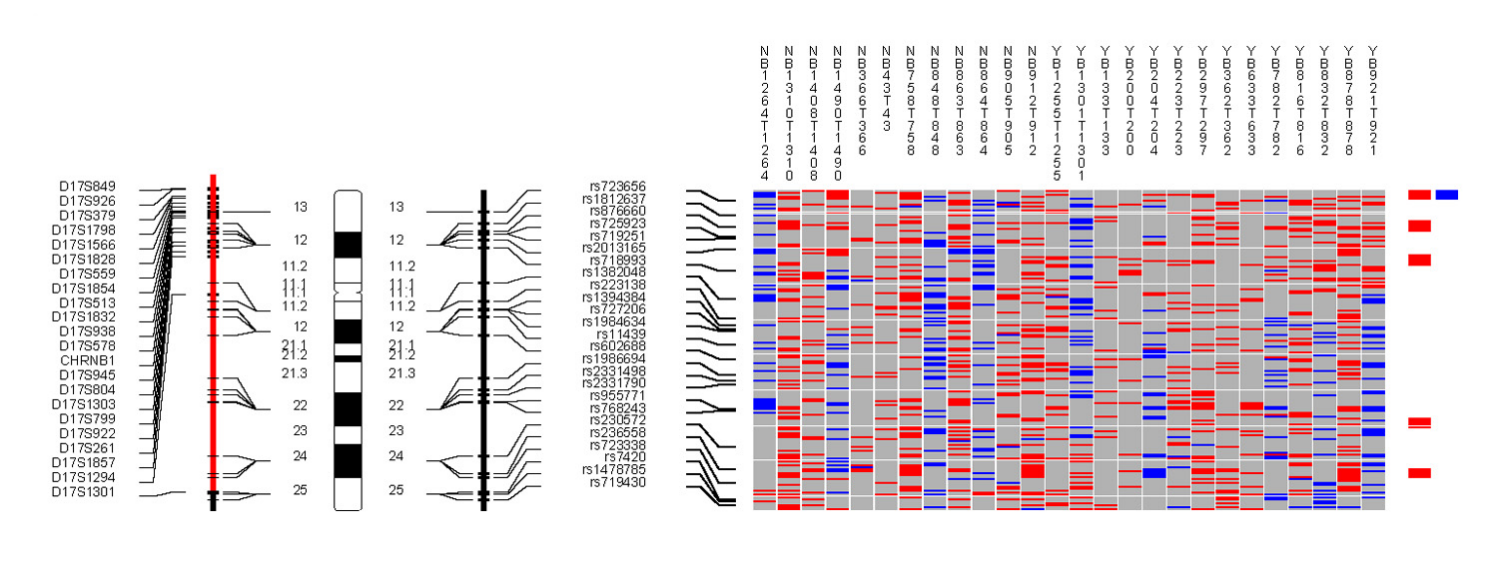
Chromosome 17. Each column in the picture represents an individual case and shows genotyping in germ-line DNA and matched micro-dissected tumor; LOH is shown in red, retention in blue, and homozygous or "no call" in grey. B indicates blood DNA and T indicates tumor DNA (from matched, micro-dissected sample). To the left of the picture, columns show (from left to right): microsatellite markers, cartoon of the chromosome, and SNPs examined in the 10 K SNP chip. To the right of the picture, red bars show deletion regions (as defined from our conservative "LOH/Model A"), blue bars show regions with CNA losses (from CNAT), and green bars show regions with CNA gains (from CNAT).

Examples of the whole genome profiles for regions on chromosome arms 9p/q, 13q, and 17p are shown in Figures 1, 2, 3.

### Comparison of LOH and cLOH regions

Our conservative LOH/Model A detected 20 LOH regions including 125 SNPs, but our conservative cLOH/Model A detected only three LOH regions containing 30 SNPs. The detection of only three LOH regions by cLOH/Model A is not unexpected since identifying an LOH region in a sample requires the presence of multiple homozygous SNPs in a large genomic area and the chance for multiple homozygous SNPs in more than 75% of the samples is low. The three cLOH regions are all on chromosome 13q12-q13. Eleven SNPs were detected by both conservative LOH and cLOH models (LOH/Model A and cLOH/Model A) (Tables 3 and 5). Five SNPs detected by the conservative cLOH/Model A are located in two genes, *FLJ14834 *and *B3GTL*. Due to the relatively small number of LOH regions defined by cLOH/Model A, as well as the different definitions of LOH used in these two approaches, it was not possible to compare the concordance between these two methods.

Our less conservative LOH/Model B and cLOH/Model B were identical except that LOH/Model B used the matched normal controls while cLOH/Model B used pooled normal control samples. SNPs in the LOH regions from LOH/Model B totaled 2,916; 2,128 SNPs were identified in the cLOH regions from cLOH/Model B. The number of SNPs common to both LOH and cLOH models (LOH/Model B and cLOH/Model B) was 1,878, while a total of 1,038 SNPs appeared only in LOH/Model B, 250 SNPs were found only in cLOH/Model B, and 7,834 showed retention in both models. Using LOH/Model B as a standard, sensitivity/specificity for cLOH/Model B were 64% and 97%, respectively. The overall Pearson correlation coefficient between these two models was 0.69 (*P *< 0.0001). Taken together, we detected more SNP loci in LOH/Model B than cLOH/Model B, but concordance between the two methods was generally good, suggesting that the use of pooled normal control samples may be acceptable for LOH studies.

### CNA regions

**Figure 4:**
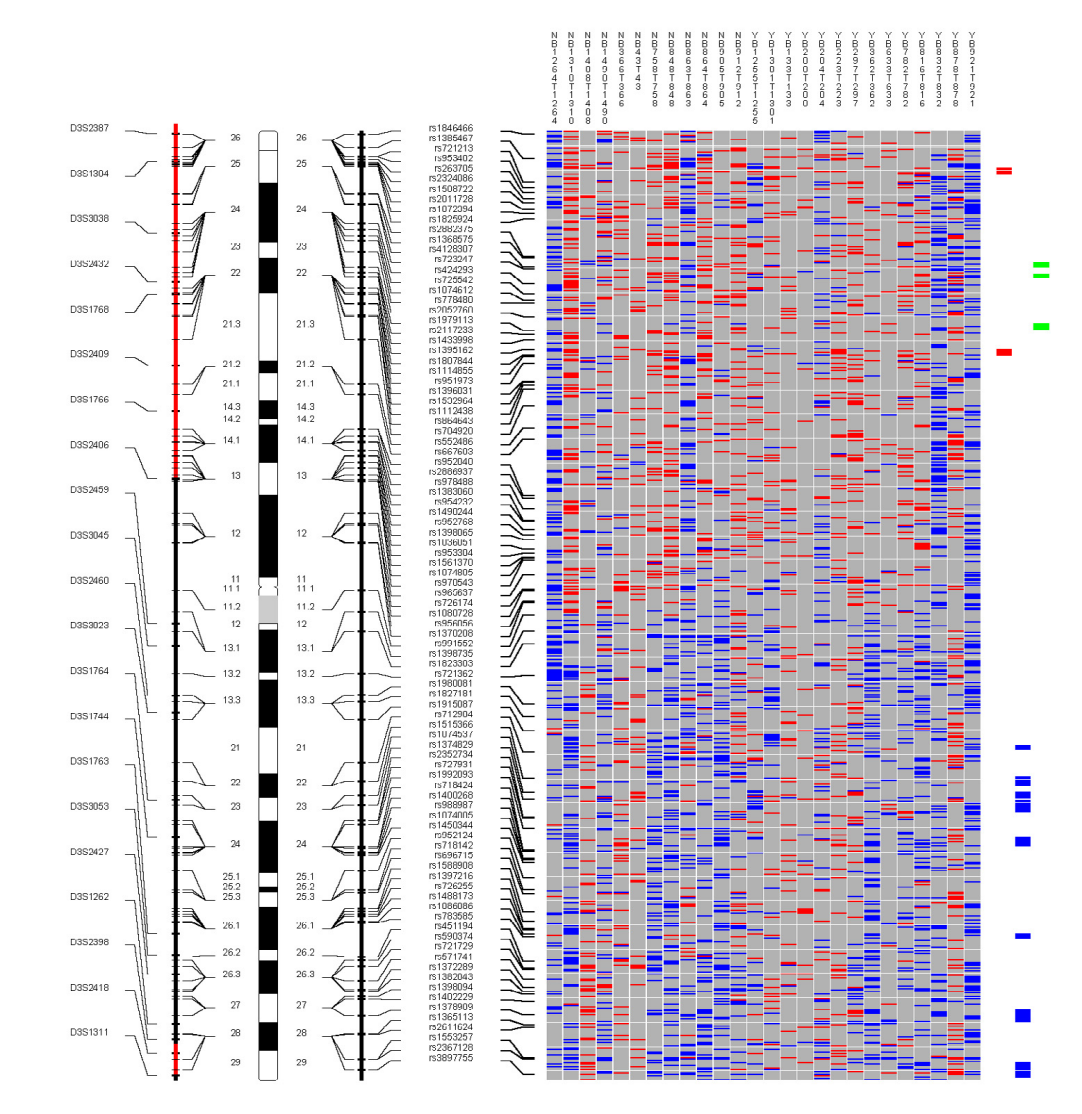
Chromosome 3. Each column in the picture represents an individual case and shows genotyping in germ-line DNA and matched micro-dissected tumor; LOH is shown in red, retention in blue, and homozygous or "no call" in grey. B indicates blood DNA and T indicates tumor DNA (from matched, micro-dissected sample). To the left of the picture, columns show (from left to right): microsatellite markers, cartoon of the chromosome, and SNPs examined in the 10 K SNP chip. To the right of the picture, red bars show deletion regions (as defined from our conservative "LOH/Model A"), blue bars show regions with CNA losses (from CNAT), and green bars show regions with CNA gains (from CNAT).

**Figure 5:**
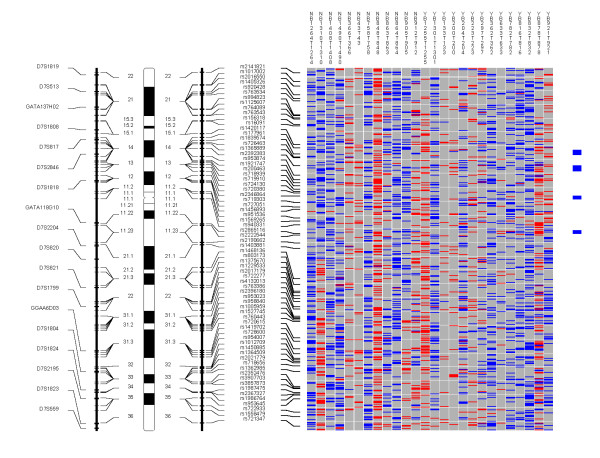
Chromosome 7. Each column in the picture represents an individual case and shows genotyping in germ-line DNA and matched micro-dissected tumor; LOH is shown in red, retention in blue, and homozygous or "no call" in grey. B indicates blood DNA and T indicates tumor DNA (from matched, micro-dissected sample). To the left of the picture, columns show (from left to right): microsatellite markers, cartoon of the chromosome, and SNPs examined in the 10 K SNP chip. To the right of the picture, red bars show deletion regions (as defined from our conservative "LOH/Model A"), blue bars show regions with CNA losses (from CNAT), and green bars show regions with CNA gains (from CNAT).

**Figure 6:**
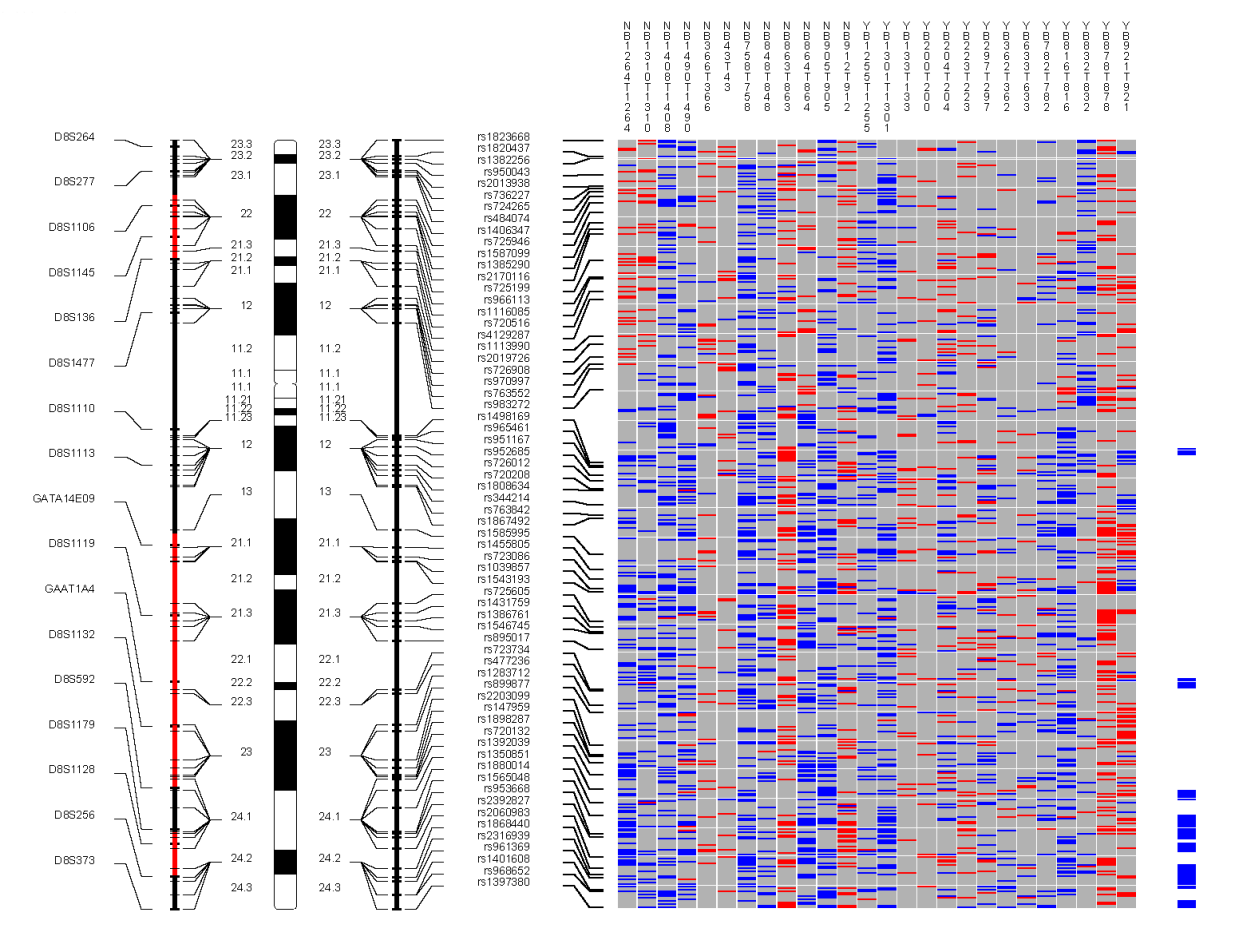
Chromosome 8. Each column in the picture represents an individual case and shows genotyping in germ-line DNA and matched micro-dissected tumor; LOH is shown in red, retention in blue, and homozygous or "no call" in grey. B indicates blood DNA and T indicates tumor DNA (from matched, micro-dissected sample). To the left of the picture, columns show (from left to right): microsatellite markers, cartoon of the chromosome, and SNPs examined in the 10 K SNP chip. To the right of the picture, red bars show deletion regions (as defined from our conservative "LOH/Model A"), blue bars show regions with CNA losses (from CNAT), and green bars show regions with CNA gains (from CNAT).

Table 7 shows 15 regions with CNA losses that were detected at *P *≤ 10^-6^. These include regions on 1p, 3p, 4q, 5q, 9p, 10p, 11p, 11q, 13q, and 18q. One-hundred and two SNPs were mapped within these regions (Table 4A). Details of the involved SNPs and genes are shown in Additional Table 3 (in additional file 1). Table 8 shows the 36 regions where significant CNA gains were identified, including eight on chromosomal arm 3q, seven on 8q, three on 7p, two on 5q, two on 14q, and two on 22q (Additional Table 4 in additional file 1). Examples of whole genome profiles of CNA regions are shown for chromosomes 3, 7, and 8 in Figures 4, 5, 6.

### Comparisons between LOH and CNA

**Figure 7:**
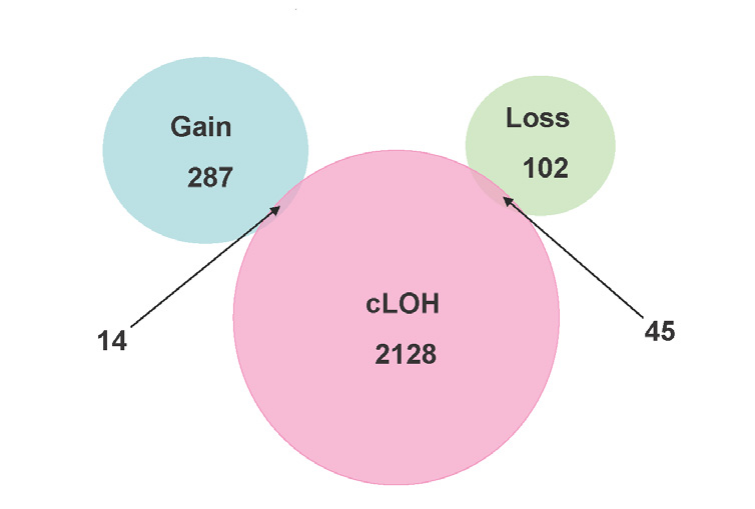
Comparison of SNPs with cLOH (from our less conservative "cLOH/Model B") and CNAs (from CNAT software using pooled DNA from normal controls).

We obtain both cLOH and CNA data when we use the pooled normal control sample reference in the CNAT software. Thus we can ask the question of whether the cLOH is associated with CNA. Our studies showed that among 2,128 SNPs identified in our less conservative cLOH/Model B, only 45 (2%) showed CNA loss and just 14 (0.7%) showed CNA gain (Figure 7). This result suggests that CNA accounts for small percent of LOH events in ESCC. LOH in cancers is commonly caused by one of three different mechanisms. The first and most common cause of LOH is mitotic recombination [3]. This mechanism doesn't change chromosome copy number, and was responsible for 97% of the LOH observed in our study. Deletion, the second cause of LOH, should result in copy number loss, and occurred in approximately two percent of LOH in our study. Finally, LOH can result from amplification of one chromosome, which should show copy number gain. This mechanism accounted for less than one percent of LOH in our study. Although chromosomal amplification occurs often, only occasionally do amplification events result in LOH, which correspond to unbalanced amplification of one chromosome. However, some studies have demonstrated concordance between LOH and CNA. For example, Wong et al found LOH associated with CNA gain at 6q12-13 in osteosarcoma [5], and Zhao et al found a link between LOH and CNA gain at 1q22-q24.1 and 1q42.13-43 in oral squamous cell carcinoma [8]. These differences might reflect genuine differences between tumor types, the lab analytic methods used, or different operative mechanisms at work.

The genome-wide LOH and chromosome copy alteration studies described in this paper can also be applied to higher density SNP chips, such as Affymetrix 100 K and 500 K SNP chips. The increased SNP density will allow even finer mapping of these genetic changes.

In summary, we performed a genome-wide study of LOH and CNA in ESCC patients using the Affymetrix 10 K SNP chip by comparing matched germ-line and tumor DNA. Our approach allowed us to extensively map both LOH and CNAs in ESCC systematically in a manner that has not heretofore been done, and produced numerous regions, genes, and SNPs that merit future exploration. This report is the first comprehensive genome-wide analysis of chromosomal imbalance (LOH and CNA) in ESCC, and the knowledge gained from this analysis will enable the development of improved strategies to prevent, diagnose, and treat ESCC patients in the future.

## Conclusion

The Affymetrix 10 K SNP chip is a valid platform to integrate analyses of loss of heterozygosity and copy number alterations. The comprehensive knowledge gained from this analysis will enable improved strategies to prevent, diagnose, and treat esophageal squamous cell carcinoma.

## Methods

### Patient selection

This study was approved by the Institutional Review Boards of the Shanxi Cancer Hospital and the US National Cancer Institute. Patients diagnosed with ESCC between 1998 and 2000 in the Shanxi Cancer Hospital in Taiyuan, Shanxi Province, People's Republic of China, and considered candidates for curative surgical resection were identified and recruited to participate in this study. None of the patients had prior therapy and Shanxi was the ancestral home for all. After obtaining informed consent, patients were interviewed to obtain information on demographic, clinical, and cancer lifestyle risk factors (smoking, alcohol drinking, and detailed family history of cancer). All patients were followed to ascertain survival status through 2003.

### Biologic specimen collection and processing

Ten milliliters of venous blood was taken from each patient prior to surgery and germ-line DNA was extracted and purified using standard methods. Tumor and adjacent normal tissue obtained during surgery were either fixed in ethanol and embedded in paraffin, or snap frozen in liquid nitrogen and stored in a freezer at -80°C until used. Slides were stained with H&E to distinguish tumor from normal epithelium, and tumor cells were micro-dissected under light microscopy using either laser capture micro-dissection (LCM) (for paraffin-embedded samples) or manual dissection (for frozen samples). All micro-dissections were performed by a pathologist (NL) and a trained post-doctoral fellow (HS). Extraction of LCM DNA was previously described [17,23]. Extraction of manually micro-dissected DNA followed the protocol from the Puregene DNA Purification Tissue Kit (Cat Number D-7000A, Gentra Systems, Inc., Minneapolis, MN 55441, USA).

It is well known that using pure tumor DNA obtained by micro-dissection is key to successfully detecting chromosomal changes such as LOH and CNA. However, the 10 K chip requires amplification of DNA fragments up to 1 Kb, a particularly challenging task. It is usually difficult to obtain a high yield of DNA from alcohol- or formalin-fixed tissues, especially when using micro-dissection. In our study, the SNP call rates were much lower in micro-dissected tumor DNA from alcohol-fixed tissue than from frozen tissue (data not shown). Although the isolation of tumor DNA from ground tissue using Trizol yielded higher genotype call rates, we think that it is more important to identify LOH and CNA regions than to simply obtain higher genotype call rates. Thus, the best overall genomic characterization results can be expected from the use of micro-dissected frozen tissue.

### Affymetrix GeneChip Mapping 10 K array

The 10 K SNP array provides comprehensive coverage of the genome for genotyping studies. Each array contained 11,555 bi-allelic polymorphic SNPs randomly distributed throughout the genome, except for the Y chromosome. The median physical distance between SNPs is approximately 105 kb, and the mean distance between SNPs is 210 kb. The average heterozygosity for these SNPs is 0.37, with an average minor allele frequency of 0.25. The algorithm used to make genotype calls was previously described by Affymetrix [24,25]. DNA samples were assayed according to the protocol (GeneChip Mapping Assay manual) supplied by Affymetrix, Inc. (Santa Clara, CA) as previously described [25,26]. The 10 K SNP arrays were scanned with the Affymetrix GeneChip Scanner 3000 using GeneChip Operating System 1.2 (GCOS) (Affymetrix). Data files were generated automatically. Genotype assignments (ie, calls) were made automatically by GeneChip DNA Analysis Software 3.0 (GDAS) (Affymetrix). The genetic map used in the analysis was obtained from GeneChip Mapping 10 K library files: Mapping 10K_Xba131. "Signal Detection Rate" is the percentage of SNPs that passed the discrimination filter. "Call Rate" is the percentage of SNPs called on the array. Genotype calls are defined as AA, AB, or BB; "no call" means the SNP for that sample did not pass the discrimination filter and was excluded from further evaluation in the present study.

### Data analysis

Since patient-matched normal DNA is not always available as a reference for high-resolution allelotyping, we evaluated LOH using two different methods: first, we used patient-matched normal DNA as the reference (the traditional approach); and second, we assessed whether it was possible to instead use a pool of normal control samples as the reference, as is done with the chromosome Copy Number Analysis Tool 2.0 software (CNAT) from Affymetrix.

In the first method, LOH was defined in a traditional manner as a change in genotyping call from heterozygosity (AB) in the germ-line DNA, to homozygosity (AA or BB) in the matched micro-dissected tumor DNA (all calls from GDAS). In the second method, LOH was also defined as a change in genotyping from "normal" to tumor, however, "normal" here was defined based on data already present in the Affymetrix CNAT software from prior testing of 100 ethnically-diverse normal reference subjects [27]. LOH in the second method was based on a comparison of a track of contiguous SNPs in tumor to the analogous track of contiguous SNPs in the "normal" population DNA. Since the "normal" DNA here includes not just one but 100 individuals, the state of these SNPs (ie, whether they are heterozygous or homozygous) was inferred statistically as a likelihood estimation with confidence calculated from a binomial distribution of the observed state of these SNPs in this normal population. A contiguous run of homozygous SNPs in tumor where these SNPs are heterozygous in the "control" suggests LOH in the region spanning the SNPs. Hence, no germ-line DNA data from cases was used for this second analysis. We refer to this LOH as "cLOH" to distinguish it from our more traditional analysis approach using paired normal and tumor samples and to indicate that it utilized a common control pool of normal DNA generated by CNAT. The threshold for statistical significance used in CNAT was *P *≤ 10^-6 ^as recommended by Affymetrix [27].

We combined the LOH results from a cluster of SNPs in a genetic locus to define a deletion region. We defined these deletion regions in several ways to permit comparison with the existing scientific literature as well as to make comparisons within our own study using different reference groups as noted above. We first used a definition that permitted comparisons with most of the existing published literature. We constrained the SNPs we considered here to require that: (i) SNPs have a call in ≥ 50% of the normal DNA samples; (ii) there be a minimum of three informative (heterozygous) normal samples; and (iii) the SNPs be mappable to NCBI Build 35.1. Non-random allelic loss was defined as LOH frequency ≥ 50% at a given locus, while random allelic loss represented LOH frequency <50% at a locus. Using these constraints for the SNPs evaluated, we defined deletion regions very conservatively by requiring that the deletion regions have five or more contiguous SNPs which showed ≥ 75% LOH. Uninformative SNPs in the regions of LOH were excluded from consideration in this analysis. Thus, a region of LOH separated from a second region of LOH by only uninformative SNPs would be combined into one large LOH region. We labeled this conservative, traditional approach "LOH/Model A".

The second approach we took (labeled "LOH/Model B") was very similar to LOH/Model A in that we used the same constraints on the SNPs noted above, but we were less conservative in our requirement for the percent of the deletion region which showed LOH – only ≥ 50% LOH frequency (instead of ≥ 75%) among the SNPs was required to be classified as a deletion region. To enable comparability with data from the cLOH approach described above, we also adopted different guidance regarding how we treated homozygous SNPs in these putative LOH regions defined by the traditional approach. In CNAT, a contiguous track of homozygous SNPs are required to designate a region as having LOH. When a homozygous SNP is located between two SNPs where one or both of the adjacent SNPs showed retention of heterozygosity, the homozygous SNP was considered retained. Otherwise, it was treated as LOH.

We also developed two models using exclusively data from the cLOH approach with the CNAT-generated pooled controls. The first used the conservative definition described above for LOH/Model A of a ≥ 75% LOH requirement to declare a deletion region, and also treated homozygous SNPs in deletion regions in accord with the CNAT algorithm described above; we termed this "cLOH/Model A". Although the level of LOH required is the same for LOH/Model A and cLOH/Model A, direct comparisons between them are not possible because of the different algorithms used to treat uninformative SNPs in deletion regions. The second approached loosened the LOH requirement to ≥ 50% to declare a deletion region (as with LOH/Model B above), also used the CNAT algorithm for homozygous SNP calls in deletion regions, and was termed "cLOH/Model B".

Individual SNP copy numbers and chromosomal regions with gains or losses were also determined by evaluation with CNAT based on the SNP hybridization signal intensity data from the experimental sample relative to intensity distributions derived from the previously described reference set containing over 100 normal individuals [27]. *P*-values were log_10_-transformed and plotted along the corresponding chromosome; values were considered significant at *P *≤ 10^-6^.

We further defined CNA-gain regions as regions where five or more contiguous SNPs showed copy number gain in at least 50% of cases, and the *P*-value for the difference from the reference was ≤ 10^-6^. Similarly, CNA-loss regions were defined as regions where five or more contiguous SNPs showed copy number loss in at least 50% of cases, and the *P*-value for the difference from the reference was ≤ 10^-6^.

## Abbreviations

SNP, single nucleotide polymorphism; ESCC, esophageal squamous cell carcinoma; LOH, loss of heterozygosity; CNA, copy number alteration; LCM, laser-capture micro-dissection; CNAT, copy number analysis tool; GCOS, GeneChip Operating System; GDAS, GeneChip DNA Analysis Software; cLOH, LOH determined by comparing patient tumor DNA with common control pool of DNA; NCBI, National Center for Biotechnology Information

## Authors' contributions

NH conceived of study, oversaw laboratory and statistical analysis, and drafted the manuscript. CW, NL, and HS performed the laboratory analyses and conducted initial statistical analyses. YH and HHY directed final statistical analyses. L-HK and Q-HW oversaw the data and sample collection procedures for the study. AMG conceived of the study, participated in statistical analyses, and revised the manuscript. KHB provided general statistical and scientific guidance for the study. ME-B conceived of the study, aided in interpretation of the data, and revised the manuscript. PRT conceived of the study, oversaw laboratory and statistical analyses, revised the manuscript, and obtained funding. MPL conceived of the study, oversaw statistical analyses, and revised the manuscript. All authors read and approved the final manuscript.

## Supplementary Material

Additional File 1 **Additional tables. Additional tables show more details of deletion regions and copy number alteration loss and gain regions**. ADDITIONAL TABLE 1. Deletion regions from the less conservative "LOH/Model B" (detailed). ADDITIONAL TABLE 2. Deletion regions from the less conservative "cLOH/Model B" (detailed). ADDITIONAL TABLE 3. Copy number alteration loss regions from CNAT (detailed). ADDITIONAL TABLE 4. Copy number alteration gain regions from CNAT (detailed). Click here for file

## Figures and Tables

**Table 1:** Demographic, risk factor, clinical characteristics, and LOH frequency of ESCC patients (N = 26)

**No**.	**ID**	**Age/sex**	**Tumor location**	**Tumor stage/grade**	**Metastasis**	**FH of UGI cancer (Y/N)**	**Tobacco use (Y/N)**	**Alcohol use (Y/N)**	**Pickled vegetables (Y/N)**	**Survival status**	**Survival months**	LOH* (blood DNA reference)	LOH* (normal esophagus reference)
1	SHE0043	72/M	lower	3/II	Y	N	Y	N	N	deceased	29.6	0.951	
2	SHE0133	55/M	lower	1/III	Y	Y	Y	N	N	deceased	36.5	0.907	
3	SHE0200	46/M	middle	1/III	Y	Y	Y	Y	Y	deceased	45	0.875	
4	SHE0204	51/M	middle	2/III	N	Y	Y	N	N	alive	64.9	0.345	
5	SHE0223	60/M	middle	2/III	N	Y	N	N	Y	deceased	34.8	0.807	
6	SHE0297	64/M	middle	4/III	Y	Y	Y	Y	N	deceased	6.9	0.596	
7	SHE0362	52/M	middle	2/I	Y	Y	Y	Y	Y	alive	58.1	0.236	
8	SHE0366	46/M	lower	3/II	Y	N	Y	Y	N	deceased	26.2	0.849	
9	SHE0633	63/M	lower	3/III	N	Y	Y	N	Y	deceased	26.5	0.635	
10	SHE0758	54/M	lower	3/III	N	N	Y	N	N	deceased	6.6	0.267	
11	SHE0782	49/F	middle	2/II	Y	Y	N	N	Y	deceased	29.6	0.211	
12	SHE0816	58/M	upper	3/III	N	Y	Y	Y	Y	deceased	30.7	0.262	
13	SHE0832	52/M	middle	3/II	N	Y	Y	Y	Y	alive	42.5	0.215	0.219
14	SHE0848	62/F	middle	3/II	N	N	Y	N	Y	alive	39.0	0.384	
15	SHE0863	51/M	upper	3/II	N	N	Y	Y	Y	alive	39.4	0.567	
16	SHE0864	55/M	middle	4/II	Y	N	Y	Y	Y	deceased	10.8	0.204	0.197
17	SHE0878	67/M	lower	3/I	N	Y	Y	Y	Y	deceased	2.2	0.806	
18	SHE0905	58/F	middle	3/II	N	N	N	N	Y	alive	35.5	0.188	
19	SHE0912	45/M	middle	3/II	Y	N	Y	N	Y	deceased	31.2	0.747	
20	SHE0921	64/M	lower	3/III	N	Y	Y	Y	Y	deceased	13.2	0.246	
21	SHE1255	61/M	middle	2/II	Y	Y	N	Y	Y	deceased	20.0	0.567	
22	SHE1264	68/M	middle	3/II	Y	N	Y	Y	N	deceased	16.2	0.27	0.267
23	SHE1301	64/M	middle	3/II	Y	Y	N	N	Y	alive	23.2	0.233	
24	SHE1310	55/F	middle	2/II	Y	N	N	N	Y	unknown	-	0.593	
25	SHE1408	63/M	middle	3/II	N	N	Y	Y	N	deceased	23.3	0.224	
26	SHE1490	52/M	middle	3/II	N	N	Y	Y	Y	alive	20.0	0.3	0.287

**Table 2:** LOH distribution by chromosomal arm

**Chr arm**	**LOH in ESCC (n = 26)**	**Chr arm**	**LOH in ESCC (n = 26)**
1p	0.282 (563/1998)	11p	0.393 (444/1130)
1q	0.211 (377/1787)	11q	0.372 (591/1590)
2p	0.301 (561/1861)	12p	0.259 (149/576)
2q	0.331 (782/2363)	12q	0.265 (496/1874)
3p	0.576 (955/1658)	13q	0.682 (1300/1907)
3q	0.287 (482/1677)	14q	0.461 (846/1836)
4p	0.602 (509/846)	15q	0.568 (845/1487)
4q	0.645 (1424/2208)	16p	0.285 (148/520)
5p	0.316 (286/906)	16q	0.338 (213/631)
5q	0.516 (1321/2560)	17p	0.755 (246/326)
6p	0.445 (545/1226)	17q	0.673 (450/669)
6q	0.417 (981/2351)	18p	0.409 (142/347)
7p	0.331 (398/1201)	18q	0.481 (513/1067)
7q	0.348 (539/1551)	19p	0.389 (58/149)
8p	0.433 (331/764)	19q	0.442 (184/416)
8q	0.313 (550/1759)	20p	0.318 (208/655)
9p	0.717 (867/1209)	20q	0.335 (178/532)
9q	0.715 (714/999)	21q	0.391 (339/866)
10p	0.367 (314/855)	22q	0.349 (157/450)
10q	0.366 (694/1897)		

**Average LOH = 0.425**			

**Table 3:** Deletion regions from the conservative "LOH/Model A"

**Deletion region no**.	**Chr**	**Cytoband(s)**	**Start (Mb)**	**End (Mb)**	**Size (Mb)**	**No. SNPs in each deletion region**	Genes in the deletion regions*
1	3	3p26	4.34	5.26	0.92	5	*SUMF1 MRPS10P2 LOC401048 **ITPR1 **BHLHB2 LOC442073 ARL10C EDEM1*
2	3	3p24-p22	31.02	33.39	2.37	5	*LOC339897 LOC391524 SIMP OSBPL10 LOC344787 KIAA0089 LOC402126 CKLFSF8 CKLFSF7 KKLFSF6*
							*LOC285260 DNCLI1 LOC391526 CNOT10 LOC389101 LOC389102 LOC131405 CCR4 GLB1CRTAP*
							KIAA0527 FBXL2 UBP1*
3	4	4p15.3	9.9	9.91	0.01	5	NA
4	4	4q13	72.58	74.06	1.48	6	*SLC4A4 GC LOC441023 GPR74 ADAMTS3 **LOC391669 FLJ1305***
5	4	4q22	86.78	88.62	1.84	7	*ARHGAP24 MAPK10 PTPN13 SOAT LOC401142 MGC26744 LOC260422 **MLLT2 **KLHL8 GAPDL4*
							***FAM13A1 **LOC441028 SCDR9*
6	4	4q22	90.72	94.98	4.26	6	*SNCA MMRN1 MGC48628 TMSL3 **LOC389212 **LOC133083 **GRID2 BMPR1B***
7	9	9p23	11.19	12.05	0.86	6	NA
8	9	9p21.3	26.02	27.53	1.51	6	*LOC441390 C9orf82 PLAA LRRC19 CCDC2 **TEK **C9orf14 C9orf11 **MOBKL2B **IFNK*
9	9	9q22.1	86.09	87.21	1.12	6	*HBLD2 ZCCHC6 LOC392358 GAS1 LOC157956 FLJ45537 **KIAA1529 TMOD1 C9orf197***
10	9	9q22.3	97.08	97.42	0.34	5	*LOC389777 LOC286359 TDRD7*
11	9	9q31.3	102.8	103.35	0.53	5	*CYLC2 LOC340511 **ZNF618***
12	13	13q12.3-q13	30	31.2	1.2	13	*C13orf22 ALOX5AP LOC387917 LOC401732 LOC440132 **FLJ14834 MGC40178 HSPH1 B3GTL 13CDNA73***
13	13	13q13.1-q13.3	32	34.89	2.89	7	*PFAAP5 APRIN LOC122038 KL **STARD13 **RFC3 **NBEA DCNAMKL1***
14	13	13q13.3	37.13	39.05	1.92	8	*TRPC4 Ufm1 DKFZp686J0811 STOML3 C13orf23 LOC387921 LOC390397 **LHFP***
15	13	13q22	75.3	76.82	1.52	8	*KCTD12 BTF3L1 LOC341720 LOC390413 DHX9P CLN5 FBXL3A MYCBP2*
16	17	17p13-	0.86	4.32	3.46	5	***ABR **MRPL14P1 LOC390754 TUSC5 YWHAE CRK MYO1C SKIP PITPN MGC34680 SCARF1 RILP*
		p13.2					*PRPF8 MGC14376 FLJ33817 SERPINF2 SERPINF1 SMYD4 RPA1 RTN4RL1 DPH2L1OVCA2 HIC1 C17orf31*
							*LOC440396 SRR FLJ10534 RUTBC1 MNT LOC284009 MGC3329 PAFAH1B1 KIAA0664 GARNL OR1D4*
							*OR1D2 OR1E3P OR1G1 OR1P1P OR1A2 OR1A1OR1D3P OR3A1 LOC390756 OR3A2 OR1R1P OR1E1*
							*OR3A3 OR1E2 NYD-SP20 ASPA TRPV3 TRPV1 CARKL CTNS TAX1BP3 MGC2963 P2RX5 GSG2*
							***ITGAE **HSA277841 CAMKK1 P2RX1P2RX1 ATP2A3 **ZZEF1 **MGC32124 ANKFY1 UBE2G1 MGC29671 **LOC124976***
17	17	17p12	9.68	10.8	1.12	6	***GLP2R **RCV1 **GAS7 **RPS27AP1 MYH13 MYH8 MYH4 MYH1 MYH2 **MYH3 **SCO1 MDS006 LOC388335*
							*LOC400573*
18	17	17p12	12.52	13.45	0.93	6	***MYOCD **KIAA0672 ELAC2 **HS3ST3A1***
19	17	17q22	51.95	52.2	0.25	5	*NOG*
20	17	17q24	67.83	68.01	0.18	5	*LOC146795 LOC440462*

**Total no. SNPs**						**125**	

*SNPs in genes (exons, introns, or 3'- or 5'-UTRs) are shown in **bold**.

**Table 4:** Deletion regions from the less conservative "LOH/Model B"

**Chr arm**	**No. deletion regions in each chr arm**	**Cytoband(s)**	**Total no. SNPs in each chr arm**
2q	1	2q35	6
3p	6	3p26-p25 3p25-p24.3 3p24 3p24-p14 3p14-p13 3p13	293
4p	4	4p16-p15.3 4p15.3 4p15.1-p13 4p12-q12	150
4q	5	4q21 4q21.2-q22 4q22-q31.2 4q31.2-q34 4q34-q35	486
5q	8	5q11.2-q15 5q15 5q21 5q22 5q23 (2) 5q32-q34 5q34-q35	357
6p	5	6p24-p23 6p22.3 6p22.2-p21.2 6p21.2 6p11.2-q12	75
6q	1	6q15	21
8p	4	8p23.2 8p22(3)	32
9p/q	3	9p24.3 9p24.3-p22 9p22-q33	507
10p	1	10p12.1-p11.2	5
11p	1	11p15.2	6
11q	2	11q23 11q24	20
13q	4	13q12.2-q31.1 13q31.1 13q32.1 13q32.1-q34	391
14q	10	14q11.2-q12 14q12 (2) 14q21 (3) 14q23 (2) 14q23-q24.2 14q24.3	138
15q	10	15q11.2-q14 15q14 15q14-q15 15q15-q21.1 15q21.1 15q21.3-q23 15q25 (3) 15q25-q26.2	190
17p/q	1	17p13-q25	174
18q	4	18q21.1-q21.2 18q21.2 18q22 (2)	47
21q	1	21q22.1	10
22q	1	22q12.1-q12	8

**Total no. deletion regions**	**72**	**Total no. SNPs**	**2916**

**Table 5:** Deletion regions from the conservative "cLOH/Model A"

**Deletion region no**.	**Cytoband**	**Start (Mb)**	**End (Mb)**	**Size (Mb)**	**No. SNPs in each deletion region**	SNP list*	**Gene list**
1	13q12-q13	27.4	29.3	1.9	15	rs720661 rs748253 rs952648 rs2987346 rs1414357 rs2388092	*LOC440129 CDX2 FLT3 PAN3*
						rs2388093 rs2388094 rs1986785 rs719199 rs1590501 rs724946	*FLT1 LOC341784 C13orf12 LOC283537*
						rs2388094 rs1986785 rs719199 rs1590501 rs724946 rs1409504	*CYP51P2 KIAA0774 SLC7A1*
						rs1409506 rs1073230	
2	13q13	29.6	30	0.4	7	rs1073229 rs1570613 rs1331697 rs1831549	*KATNAL1 PRDX2P1 UBCH7N2 HMGB1*
						rs1343946* rs1343945 rs1854953	*FLJ14834*
3	13q13	30.3	30.5	0.2	8	**rs2007622 *rs876540 rs912603 *rs2056443 rs13080**	*LOC387917 LOC401732 LOC440132*
						**rs1409379 rs1328950 rs1328949**	*FLJ14834 MGC40178 B3GTL*

**Total no. SNPs**					**30**		

*Italicized SNPs are in genes; **bolded **SNPs are also found in the 20 deletion regions of the conservative LOH/Model A.

**Table 6:** Deletion regions from the less conservative "cLOH/Model B"

**Chr arm**	**No. regions in each chr arm**	**Cytoband(s)**	**Total no. SNPs in each chr arm**
2q	2	2q34-q35 2q35	33
3p	7	3p26 (2) 3p26-p25 3p21.32-p2.2	182
4p	4	4p16.3-p15.3 4p15.3 4p12 4p12-q12	100
4q	14	4q13 (2) 4q13-4q22 4q26 4q27 4q27-q28 4q28	377
		4q28-q32 4q32 4q32-q33 4q33-q34 4q34	
5q	15	5q11.2 5q11.2-q12 5q12 5q13 5q13-q15 5q15 5q15-q21	386
		5q21-q22 5q22 (2) 5q23.1 5q23.1-q23 5q32-q34 5q34-q35 5q35	
8p	2	8p22 (2)	13
9p	5	9p24.3-p23 9p23-p21 9p21.3-p21.1 9p21-p13 9p13-q22	367
9q	2	9q22.2-q22.3 9q22.3-q33	147
13q	7	13q12.2-q14 13q14-q21.1 13q21.1-q21.33	365
		13q21.33-q31.1 13q31.3-q32.1 13q32.1-q33 13q33	
17p	2	17p13-p12 17p12-q21	96
17q	3	17q21-q22 17q22-q24 17q24	57
21q	1	21q22.2	5

**Total no. deletion regions**	**64**	**Total no. SNPs**	**2128**

**Table 7:** Regions with copy number alteration loss from CNAT

**Chr arm**	**No. CNA loss regions in each chr arm**	**Cytoband(s)**	**Total no. SNPs in each chr arm**
1p	1	1p13.3	5
3p	3	3p24 3p25-p24.3 3p25	19
4q	1	4q28	5
5q	2	5q14-q15 5q33	23
9p	1	9p21.3	8
10p	1	10p12-p12.1	7
11p	1	11p15.5	5
11q	2	11q21 (2)	10
13q	2	13q12.2-q12 13q14	14
18q	1	18q22	6

**Total no. loss regions**	**15**	**Total no. SNPs**	**102**

**Table 8:** Regions with copy number alteration gain from CNAT

**Chr arm**	**No. CNA gain regions in each chr arm**	**Cytoband(s)**	**Total no. SNPs in each chr arm**
1p	1	1p36.13	5
1q	1	1q21.3-1q21	8
2p	1	2p14	6
2q	2	2q11.2 2q21	15
3q	8	3q21-q22 3q22-q23 3q23(2) 3q24 3q26.1 3q27 3q28	69
5p	2	5p15.2 (2)	14
5q	1	5q11.2	5
6p	1	6p25.3-p24	8
7p	3	7p14 (2) 7p12	24
7q	1	7q11.21-q11.22	5
8q	7	8q24.2 (2) 8q24.1 (3) 8q22 8q11.2	66
9q	1	9q31-q31.3	7
14q	2	14q21-q22 14q22-q23	16
17p	1	17p13.1-p13.2	7
18p	1	18p11.31	8
20p	1	20p11.1-q11.1	13
22q	2	22q12.1 22q13.1	11

**Total no. gain regions**	**36**	**Total no. SNPs**	**287**
