# Supplementary material for: Genome-wide loss of heterozygosity and copy number alteration in esophageal squamous cell carcinoma using the Affymetrix GeneChip Mapping 10 K array
Source: BMC Genomics. 2006 Nov 29;7:299. doi: 10.1186/1471-2164-7-299 (PMC1687196; doi:10.1186/1471-2164-7-299)
Supplement: Additional File 1 — Additional tables. Additional tables show more details of deletion regions and copy number alteration loss and gain regions. ADDITIONAL TABLE 1. Deletion regions from the less conservative "LOH/Model B" (detailed). ADDITIONAL TABLE 2. Deletion regions from the less conservative "cLOH/Model B" (detailed). ADDITIONAL TABLE 3. Copy number alteration loss regions from CNAT (detailed). ADDITIONAL TABLE 4. Copy number alteration gain regions from CNAT (detailed). [file 1471-2164-7-299-S1.doc]

| **ADDITIONAL tABLE 1. Deletion regions from the less conservative "LOH/Model B" (detailed)** | | | | | | |
| --- | --- | --- | --- | --- | --- | --- |
|  |  |  |  |  |  |  |
| Deletion | Chr | Cytoband | Start | End | Size | No. |
| region no. |  |  | (Mb) | (Mb) | (Mb) | SNPs |
| 1 | 2 | 2q35 | 220.23 | 221.02 | 0.79 | 6 |
| 2 | 3 | 3p26 -p25 | 2.67 | 14.85 | 12.18 | 93 |
| 3 | 3 | 3p25 -p24 | 15.74 | 22.5 | 6.76 | 31 |
| 4 | 3 | 3p24 | 29.52 | 30.17 | 0.65 | 6 |
| 5 | 3 | 3p24-p14 | 31.4 | 65.55 | 34.15 | 144 |
| 6 | 3 | 3p14-p13 | 67.46 | 68.71 | 1.25 | 7 |
| 7 | 3 | 3p13 | 70.76 | 74.03 | 3.27 | 12 |
| 8 | 4 | 4p16- p15.3 | 5.8 | 11.6 | 5.8 | 29 |
| 9 | 4 | 4p15.3 | 15.16 | 17.95 | 2.79 | 20 |
| 10 | 4 | 4p15.1-p13 | 26.15 | 44.43 | 18.28 | 72 |
| 11 | 4 | 4p12-q12 | 47.77 | 59.94 | 12.17 | 29 |
| 12 | 4 | 4q12-4q21 | 60.46 | 76.57 | 16.11 | 76 |
| 13 | 4 | 4q21-q22 | 77.18 | 94.98 | 17.8 | 88 |
| 14 | 4 | 4q22-q31.2 | 97 | 146.74 | 49.74 | 187 |
| 15 | 4 | 4q31.2-q34 | 148.21 | 177.31 | 29.1 | 123 |
| 16 | 4 | 4q34 -q35 | 184.13 | 187.53 | 3.4 | 12 |
| 17 | 5 | 5q11.2-q15 | 56.89 | 89.28 | 32.39 | 138 |
| 18 | 5 | 5q15 | 95.12 | 96.41 | 1.29 | 7 |
| 19 | 5 | 5q21 | 98.59 | 109.1 | 10.51 | 53 |
| 20 | 5 | 5q22 | 114.19 | 115.66 | 1.47 | 8 |
| 21 | 5 | 5q23.1 | 121.08 | 125.56 | 4.48 | 33 |
| 22 | 5 | 5q23 | 127.87 | 129.88 | 2.01 | 7 |
| 23 | 5 | 5q32-q34 | 149.13 | 161.88 | 12.75 | 75 |
| 24 | 5 | 5q34-q35 | 164.54 | 173.36 | 8.82 | 36 |
| 25 | 6 | 6p24-p23 | 8.54 | 12.33 | 3.79 | 8 |
| 26 | 6 | 6p22.3 | 21.09 | 23.44 | 2.35 | 9 |
| 27 | 6 | 6p22.2-p21.2 | 27.39 | 32.79 | 5.4 | 26 |
| 28 | 6 | 6p21.2 | 36.13 | 41.21 | 5.08 | 20 |
| 29 | 6 | 6p11.2-q12 | 54.94 | 61.84 | 6.9 | 12 |
| 30 | 6 | 6q15 | 86.47 | 90.8 | 4.33 | 21 |
| 31 | 8 | 8p23.2 | 7.16 | 8.02 | 0.86 | 7 |
| 32 | 8 | 8p22 | 13.96 | 14.64 | 0.68 | 7 |
| 33 | 8 | 8p22 | 14.74 | 15.94 | 1.2 | 12 |
| 34 | 8 | 8p22 | 16.62 | 17.29 | 0.67 | 6 |
| 35 | 9 | 9p24.3 | 2.39 | 2.99 | 0.6 | 5 |
| 36 | 9 | 9p24.3 -p22 | 3.17 | 18.68 | 15.51 | 125 |
| 37 | 9 | 9p22-q33 | 19.61 | 121.39 | 101.78 | 377 |
| 38 | 10 | 10p12.1-p11.2 | 29.38 | 30 | 0.62 | 5 |
| 39 | 11 | 11p15.2 | 11.77 | 12.83 | 1.06 | 6 |
| 40 | 11 | 11q23 | 114.38 | 117.5 | 3.12 | 11 |
| 41 | 11 | 11q24 | 121.23 | 122.72 | 1.49 | 9 |
| 42 | 13 | 13q12.2 -q31.1 | 21.27 | 79.46 | 58.19 | 308 |
| 43 | 13 | 13q31.1 | 87.26 | 87.35 | 0.09 | 5 |
| 44 | 13 | 13q32.1 | 93.8 | 94.23 | 0.43 | 6 |
| 45 | 13 | 13q32.1-q34 | 94.42 | 109.48 | 15.06 | 72 |
| 46 | 14 | 14q11.2 -q12 | 21.42 | 28.1 | 6.68 | 28 |
| 47 | 14 | 14q12 | 29.35 | 30.65 | 1.3 | 7 |
| 48 | 14 | 14q12 | 31.58 | 32.12 | 0.54 | 8 |
| 49 | 14 | 14q21 | 37.72 | 41.18 | 3.46 | 19 |
| 50 | 14 | 14q21 | 43.14 | 45.27 | 2.13 | 6 |
| 51 | 14 | 14q21 | 46.98 | 49.83 | 2.85 | 7 |
| 52 | 14 | 14q23 | 56.34 | 58.91 | 2.57 | 15 |
| 53 | 14 | 14q23 | 61.31 | 64.58 | 3.27 | 12 |
| 54 | 14 | 14q23-q24.2 | 65.08 | 71.21 | 6.13 | 30 |
| 55 | 14 | 14q24.3 | 76.46 | 77.01 | 0.55 | 6 |
| 56 | 15 | 15q11.2-q14 | 28.23 | 29.02 | 0.79 | 5 |
| 57 | 15 | 15q14 | 29.26 | 30.22 | 0.96 | 5 |
| 58 | 15 | 15q14-q15 | 33.93 | 40.96 | 7.03 | 21 |
| 59 | 15 | 15q15-q21.1 | 42.22 | 43.6 | 1.38 | 10 |
| 60 | 15 | 15q21.1 | 44.82 | 46.74 | 1.92 | 12 |
| 61 | 15 | 15q21.3-q23 | 52.72 | 71.78 | 19.06 | 57 |
| 62 | 15 | 15q25 | 74.89 | 75.34 | 0.45 | 7 |
| 63 | 15 | 15q25 | 76.69 | 78.47 | 1.78 | 7 |
| 64 | 15 | 15q25 | 78.79 | 84.26 | 5.47 | 26 |
| 65 | 15 | 15q25-q26.2 | 85.9 | 93.1 | 7.2 | 40 |
| 66 | 17 | 17p13.2-q25 | 3.87 | 72.82 | 68.95 | 174 |
| 67 | 18 | 18q21.1-q21.2 | 46.21 | 47.07 | 0.86 | 5 |
| 68 | 18 | 18q21.2 | 47.62 | 54.52 | 6.9 | 31 |
| 69 | 18 | 18q22 | 55.96 | 57.66 | 1.7 | 6 |
| 70 | 18 | 18q22 | 59.06 | 59.56 | 0.5 | 5 |
| 71 | 21 | 21q22.1 | 34.11 | 35.51 | 1.4 | 10 |
| 72 | 22 | 22q12.1-q12 | 25.3 | 27.92 | 2.62 | 8 |
| **Total no. SNPs** | | | | | | **2916** |

| **ADDITIONAL TABLE 2. Deletion regions from the less conservative “cLOH/Model B" (detailed)** | | | | | | |
| --- | --- | --- | --- | --- | --- | --- |
|  |  |  |  |  |  |  |
| Deletion | Chr | Cytoband | Start | End | Size | No. |
| region no. |  |  | (Mb) | (Mb) | (Mb) | SNPs |
| 1 | 2 | 2q34-q35 | 211.9 | 218.8 | 6.9 | 28 |
| 2 | 2 | 2q35 | 219 | 219.8 | 0.8 | 5 |
| 3 | 3 | 3p26 | 1.1 | 5.3 | 4.2 | 36 |
| 4 | 3 | 3p26 | 5.5 | 7.3 | 1.8 | 20 |
| 5 | 3 | 3p26-p25 | 8.5 | 13.1 | 4.6 | 23 |
| 6 | 3 | 3p22-p21.2 | 36.6 | 53.6 | 17 | 49 |
| 7 | 3 | 3p21.2 | 54.1 | 57.5 | 3.4 | 20 |
| 8 | 3 | 3p14 | 58.8 | 59.9 | 1.1 | 12 |
| 9 | 3 | 3p14 | 62.9 | 65.8 | 2.9 | 22 |
| 10 | 4 | 4p16.3-p15.3 | 4.2 | 14.2 | 10 | 55 |
| 11 | 4 | 4p15.3 | 14.7 | 15.7 | 1 | 11 |
| 12 | 4 | 4p12 | 45.1 | 47 | 1.9 | 7 |
| 13 | 4 | 4p12-q12 | 47.8 | 58.4 | 10.6 | 27 |
| 14 | 4 | 4q13 | 68.6 | 70.1 | 1.5 | 9 |
| 15 | 4 | 4q13 | 70.1 | 74.2 | 4.1 | 18 |
| 16 | 4 | 4q13-q22 | 74.7 | 93.3 | 18.6 | 98 |
| 17 | 4 | 4q22-q26 | 97.4 | 114.8 | 17.4 | 49 |
| 18 | 4 | 4q26 | 115 | 118.2 | 3.2 | 12 |
| 19 | 4 | 4q26-q27 | 119.1 | 124.8 | 5.7 | 23 |
| 20 | 4 | 4q27 | 125.2 | 126 | 0.8 | 9 |
| 21 | 4 | 4q27-q28 | 126.5 | 127 | 0.5 | 6 |
| 22 | 4 | 4q28 | 132.8 | 136 | 3.2 | 13 |
| 23 | 4 | 4q28-q32 | 144.5 | 156.8 | 12.3 | 53 |
| 24 | 4 | 4q32 | 157.1 | 162.9 | 5.8 | 20 |
| 25 | 4 | 4q32-q33 | 168.5 | 172.6 | 4.1 | 17 |
| 26 | 4 | 4q33-q34 | 172.7 | 180.6 | 7.9 | 45 |
| 27 | 4 | 4q34 | 181.7 | 182.2 | 0.5 | 5 |
| 28 | 5 | 5q11.2 | 57.3 | 57.9 | 0.6 | 6 |
| 29 | 5 | 5q11.2-q12 | 58.3 | 62 | 3.7 | 14 |
| 30 | 5 | 5q12 | 62.2 | 69.8 | 7.6 | 33 |
| 31 | 5 | 5q13 | 74.3 | 79.1 | 4.8 | 27 |
| 32 | 5 | 5q13-q15 | 79.4 | 89.5 | 10.1 | 39 |
| 33 | 5 | 5q15 | 90.7 | 94.6 | 3.9 | 11 |
| 34 | 5 | 5q15-q21 | 95.4 | 99.8 | 4.4 | 15 |
| 35 | 5 | 5q21-q22 | 100.6 | 113 | 12.4 | 63 |
| 36 | 5 | 5q22 | 113.7 | 115.8 | 2.1 | 13 |
| 37 | 5 | 5q22 | 117 | 117.9 | 0.9 | 8 |
| 38 | 5 | 5q23.1 | 121.2 | 121.9 | 0.7 | 8 |
| 39 | 5 | 5q23.1-q23 | 122.1 | 123 | 0.9 | 9 |
| 40 | 5 | 5q32-q34 | 148.2 | 162.9 | 14.7 | 81 |
| 41 | 5 | 5q34-q35 | 162.9 | 170.9 | 8 | 35 |
| 42 | 5 | 5q35 | 172.3 | 179.9 | 7.6 | 24 |
| 43 | 8 | 8p22 | 12.7 | 13.2 | 0.5 | 5 |
| 44 | 8 | 8p22 | 13.7 | 14.6 | 0.9 | 8 |
| 45 | 9 | 9p24.3-p23 | 2.1 | 6.4 | 4.3 | 30 |
| 46 | 9 | 9p23-p21 | 6.7 | 20.9 | 14.2 | 117 |
| 47 | 9 | 9p21-p21.1 | 21.2 | 28.4 | 7.2 | 59 |
| 48 | 9 | 9p21-p13 | 29.6 | 57.8 | 28.2 | 45 |
| 49 | 9 | 9p13-q22 | 59.2 | 89.7 | 30.5 | 116 |
| 50 | 9 | 9q22.2-q22.3 | 90 | 97.3 | 7.3 | 31 |
| 51 | 9 | 9q22.3-q33 | 97.3 | 123.7 | 26.4 | 116 |
| 52 | 13 | 13q12.2-q14 | 21.3 | 42.5 | 21.2 | 120 |
| 53 | 13 | 13q14 -q21.1 | 42.7 | 58 | 15.3 | 75 |
| 54 | 13 | 13q21.1-q21.33 | 58.3 | 68.6 | 10.3 | 41 |
| 55 | 13 | 13q21.33-q31.1 | 68.9 | 79.3 | 10.4 | 68 |
| 56 | 13 | 13q31.3 -q32.1 | 91.7 | 94.1 | 2.4 | 16 |
| 57 | 13 | 13q32.1-q33 | 94.4 | 101.1 | 6.7 | 25 |
| 58 | 13 | 13q33 | 101.2 | 104.6 | 3.4 | 20 |
| 59 | 17 | 17p13-p12 | 0.9 | 6.2 | 5.3 | 10 |
| 60 | 17 | 17p12-q21 | 8.5 | 41.4 | 32.9 | 86 |
| 61 | 17 | 17q21-q22 | 42.6 | 50.5 | 7.9 | 25 |
| 62 | 17 | 17q22-q24 | 50.9 | 61.1 | 10.2 | 21 |
| 63 | 17 | 17q24 | 63.7 | 67.2 | 3.5 | 11 |
| 64 | 21 | 21q22.2 | 38 | 38.2 | 0.2 | 5 |
| **Total no. SNPs** | | | | | | **2128** |

| **ADDITIONAL TABLE 3. Copy number alteration loss regions from CNAT (detailed)** | | | | | | | | |
| --- | --- | --- | --- | --- | --- | --- | --- | --- |
|  |  |  |  |  |  |  |  |  |
| Loss | Chr | Cytoband | Start | End | Size | No. | SNP list | Gene list |
| region no. |  |  | (Mb) | (Mb) | (Mb) | SNPs |  |  |
|  |  |  |  |  |  |  |  |  |
| 1 | 1 | 1p13.3 | 114.8 | 115.4 | 0.6 | 5 | rs2057127 rs537732 rs537692 rs950681 rs950679 | *FLJ37099 AMPD1 NRAS D1S155E FLJ21168 SYCP1 TSHB TSPAN-2* |
|  |  |  |  |  |  |  |  |  |
| 2 | 3 | 3p25 | 14.8 | 15.8 | 1 | 5 | rs294634 rs2880478 rs2061187 rs4128307 rs1947147 | *FGD5 NR2C2 MRPS25 ZFYVE20 LOC344875 CAPN7 SH3BP5 MGC24132 EAF1 COLQ HPCL2 BTD ANKRD28* |
|  |  |  |  |  |  |  |  |  |
| 3 | 3 | 3p25- 3p24.3 | 17.2 | 18.6 | 1.4 | 6 | rs723813 rs728022 rs958542 rs717793 rs717939 rs1398922 | *TBC1D5 LOC151842 LOC339862 SATB1 LOC401056 LOC131185* |
|  |  |  |  |  |  |  |  |  |
| 4 | 3 | 3p24 | 27.8 | 28.9 | 1.1 | 8 | rs2371121 rs1609729 rs2371341 rs1381392 rs1381391 rs1350867 rs2371342 rs952629 | *MGC61571 AZI2 LOC152098 FLJ33651* |
|  |  |  |  |  |  |  |  |  |
| 5 | 4 | 4q28 | 137.6 | 138.2 | 0.6 | 5 | rs898429 rs898428 rs720733 rs1946359 rs2162202 | *LOC152620* |
|  |  |  |  |  |  |  |  |  |
| 6 | 5 | 5q14- 5q15 | 84.4 | 88.7 | 4.3 | 17 | rs2115152 rs952840 rs952841 rs2410741 rs878196 rs1406407 rs3915320 rs4129235 rs40504 rs770189 rs190982 rs819350 rs1072961 rs256516 rs2222244 rs1967255 rs1967256 | *LOC402219 LOC391809 LOC441092 MGC48637 COX7C LOC389308 RASA1 CCNH MGC33214 LOC442136 MEF2C* |
|  |  |  |  |  |  |  |  |  |
| 7 | 5 | 5q33 | 155.7 | 156.2 | 0.5 | 6 | rs998997 rs1354565 rs1394605 rs1394606 rs1504934 rs256846 | *SGCD* |
|  |  |  |  |  |  |  |  |  |
| 8 | 9 | 9p21.3 | 27.3 | 27.7 | 0.4 | 8 | rs724502 rs1555453 rs763372 rs774354 rs1411381 rs1411380 rs1411379 rs1411375 | *MOBKL2B IFNK C9orf72 LOC392298* |
|  |  |  |  |  |  |  |  |  |
| 9 | 10 | 10p12.1 | 25.1 | 25.7 | 0.6 | 7 | rs1414456 rs1414457 rs1414458 rs1414459 rs951857 rs1340002 rs956088 | *PRTFDC1 C10orf63 THNSL1 GPR158* |
|  |  |  |  |  |  |  |  |  |
| 10 | 11 | 11p15.5 | 5.4 | 5.4 | 0 | 5 | rs1391613 rs1391612 rs1353736 rs1391611 rs1391610 | NA |
|  |  |  |  |  |  |  |  |  |
| 11 | 11 | 11q21 | 97.1 | 97.6 | 0.5 | 5 | rs2097160 rs1986446 rs561146 rs624607 rs625569 | NA |
|  |  |  |  |  |  |  |  |  |
| 12 | 11 | 11q21 | 99.1 | 99.3 | 0.2 | 5 | rs1389786 rs1389785 rs1389784 rs952700 rs594808 | NA |
|  |  |  |  |  |  |  |  |  |
| 13 | 13 | 13q12.2- 13q12 | 23.3 | 25.5 | 2.2 | 9 | rs2031160 rs2031161 rs720651 rs720652 rs1410281 rs1175723 rs2420130 rs718652 rs513093 | *LOC387911 FLJ46358 SPATA13 MGC48915 LOC390385 HCP33 ADPRTL1 LOC387912 LOC374491 ATP12A LOC390386 RNF17 TDRD4 CENPJ LOC390387 PABPC3 FLJ25477 MTMR6 NUPL1 LOC246717 ATP8A2 LOC387914* |
|  |  |  |  |  |  |  |  |  |
| 14 | 13 | 13q14 | 43 | 44 | 1 | 5 | rs1373904 rs717090 rs717091 rs1413081 rs1413083 | *FLJ31846 FLJ38725 LOC400126 LOC440133 LOC440134 C13orf21 LOC400128* |
|  |  |  |  |  |  |  |  |  |
| 15 | 18 | 18q22 | 62.3 | 62.7 | 0.4 | 6 | rs1373369 rs764133 rs1824484 rs491544 rs2126015 rs726523 | *CDH19* |

| **ADDITIONAL TABLE 4. Copy number alteration gain regions from CNAT (detailed)** | | | | | | | | |
| --- | --- | --- | --- | --- | --- | --- | --- | --- |
|  | | | | | | | | |
| Gain region ID | Chr | CytoBand | Start (Mb) | End (Mb) | Size (Mb) | No. SNPs | SNPList | GeneList |
| 1 | 1 | 1p36.13 | 19.2 | 19.4 | 0.2 | 5 | rs743986 rs1076624 rs230175 rs764151 rs721360 | *C1orf33 AFAR3 AKR7A3 AKR7A2 PQLC2* |
| 2 | 1 | 1q21.3 1q21 | 146.5 | 150 | 3.9 | 8 | rs1560832 rs724781 rs2335230 rs1395565 rs951241 rs951781 rs725377 rs1338685 | *LOC388692 LOC199882 FCGR1A LOC440689 LOC441906 HIST2H3A HIST2H2AA  LOC388693 HIST2H2BC HIST2H4 HIST2H2BE HIST2H2AC HIST2H2AB CGI-143  SV2A SF3B4 CRA ZA20D1 LOC441907 VPS45A CKIP-1 ANP32E CA14 APH-1A  FLJ23221 LOC148523 MRPS21 PRPF3 KIAA0460 FLJ12528 ECM1 FLJ13544 TSRC1  MCL1 ENSA GPP34R NOHMA CTSS CTSK ARNT HCP1 SETDB1 LASS2 ANXA9  FLJ11280 HTCD37 BNIPL FLJ20519 CDC42SE1 AF1Q MGC29891 SEMA6C FLJ23467  SB145 SCNM1 TMOD4 TCFL1 PIP5K1A PSMD4 KIAA1441 PIK4CB RFX5 SELENBP1  PSMB4 POGZ CGN TUFT1 SNX27 TNRC4 FLJ36032 MRPL9 OAZ3 TDRKH LOC440691  LOC339398 RORC FLJ37964 CTMP LOC391099 S100A10 LOC441908 S100A11 LOC126637  FAM16B FLJ39117 LOC388697 LOC400785 LOC400786 FLG LOC388698 C1orf10 SPRL5A  NICE-1 LEP17 SPRL6A SPRL3A LEP14 LEP13 xp33 SPRL1A LEP11 SPRL1B LEP9  SPRL4A LEP6 LEP5 LEP4 LEP3 SPRL2A LEP1 MCSP IVL SPRR4 SPRR1A SPRR3  SPRR1B SPRR2B SPRR2A LOC440692 LOC149018 LOC391102 LOR PGLYRP3 PGLYRP4  S100A9 S100A12 S100A8 S100A15 S100A7 S100A6 S100A5 S100A4 S100A3 S100A2* |
| 3 | 2 | 2p14 | 66.7 | 67.7 | 1 | 6 | rs1073981 rs1110581 rs2216925 rs726085 rs2060042 rs966441 | *LOC391381 ETAA16* |
| 4 | 2 | 2q11.2 | 97.1 | 99.4 | 2.3 | 9 | rs1561002 rs1374324 rs958790 rs953934 rs950881 rs1861229 rs2310316 rs1542601 rs726653 | *LOC391409 LOC440891 DKFZp434N062 UNQ2430 LOC442031 LOC442032 UBE3AP1  LOC442033 LOC442034 KIAA1641 LOC151320 COX5B ACTR1B RNU4P2 LOC400988  ZAP70 RW1 MGC26733 CNGA3 INPP4A UNC50 MGAT4A MGC42367 TSGA10 C2orf15  LIPT1 LOC129531 MRPL30 LYG2 LOC129530 TXNDC9* |
| 5 | 2 | 2q21 | 128.6 | 129 | 0.8 | 6 | rs2321201 rs3886664 rs1867898 rs1564935 rs1374721 rs1598279 | *UGCGL1 HS6ST1* |
| 6 | 3 | 3q21 3q22 | 129 | 132 | 2.9 | 9 | rs977683 rs1986471 rs718613 rs718614 rs718612 rs725132 rs1905441 rs907931 rs925612 | *FLJ46299 SEC61A1 RUVBL1 SELB DNAJB8 GATA2 LOC442089 GR6 RPN1 LOC442090  RAB7 LOC391574 LOC389147 ACAD9 KIAA1257 LOC401087 FLJ12057 LOC442091 GP9  RAB43 KIAA1160 ZNF9 COPG DC12 H1FX LOC339942 LOC132241 LOC90288 MBD4  WDR10 RHO H1FOO PLXND1 KIAA0779 TRH LOC401389 OR7E129P OR7E21P LOC442092  LOC440976 LOC440977 LOC440978 FLJ35880 LOC131873 PIK3R4* |
| 7 | 3 | 3q22 3q23 | 136.7 | 139 | 2.2 | 7 | rs719893 rs1515366 rs1515367 rs36117 rs2177693 rs361239 rs361238 | *PPP2R3A FLJ10546 LOC391581 PCCB STAG1 TMEM22 NCK1 MGC34923* |
| 8 | 3 | 3q23 | 139.8 | 142 | 1.9 | 6 | rs1074537 rs1074538 rs1429759 rs211587 rs952797 rs878784 | *FAIM LOC256374 PIK3CB LOC389150 FOXL2 LOC401089 LOC389151 LOC389152  BPESC1 MRPS22 COPB2 RBP2 ACTGP1 RBP1 NMNAT3 CLSTN2* |
| 9 | 3 | 3q23 | 141.9 | 143 | 0.6 | 9 | rs727763 rs2350488 rs1005706 rs953460 rs953459 rs953461 rs879716 rs1427781 rs724761 | *TRIM42 FLJ10618 SSB4 FLJ23751* |
| 10 | 3 | 3q24 | 148.3 | 150 | 1.8 | 8 | rs1403645 rs342885 rs966226 rs2018842 rs951463 rs1116440 rs1116439 rs958465 | *ZIC4 ZIC1 LOC440982 LOC344741 LOC389159 RPL38P1 AGTR1 CPB1 CPA3* |
| 11 | 3 | 3q26.1 | 166.3 | 167 | 0.2 | 6 | rs491306 rs1086086 rs721976 rs783585 rs810692 rs1656113 | *SLITRK3* |
| 12 | 3 | 3q27 | 185.3 | 187 | 2.1 | 13 | rs1983421 rs262982 rs262981 rs262980 rs1378909 rs1829515 rs2872249 rs727272 rs725656 rs2377107 rs2377256 rs721814 rs719393 | *EIF2B5 LOC440991 DVL3 AP2M1 ABCF3 LOC90113 ALG3 MGC2408 CAM-KIIN ECE2  PSMD2 EIF4G1 MGC21688 CLCN2 POLR2H THPO CHRD LOC285248 LOC391600 EPHB3  MAGEF1 LOC389182 KIAA0804 LOC285382 LOC339926 EHHADH EIF2S2P2 MAP3K13  MGC15397 LIPH SENP2 IMP-2 SFRS10 LOC344887 ETV5* |
| 13 | 3 | 3q28 | 195.5 | 198 | 2.3 | 11 | rs951016 rs1017972 rs724767 rs1986454 rs898824 rs860309 rs952481 rs952586 rs3897755 rs950257 rs2344605 | *LOC285280 LRRC15 GP5 AFURS1 LOC401106 LOC93109 FLJ11301 FLJ90022 LOC285303  FLJ35155 CENTB2 PPP1R2 LOC255812 LOC440993 MUC20 MUC4 LOC285253 LOC402157  ACK1 LOC440994 TFRC FLJ25996 ZDHHC19 OSTalpha PCYT1A MGC33212 LOC116211  KIAA0794 LOC389188 FLJ35794 LOC255798* |
| 14 | 5 | 5p15.2 | 8.4 | 8.7 | 0.3 | 8 | rs1157158 rs1448235 rs340680 rs340681 rs1448229 rs341880 rs386332 rs1501321 | NA |
| 15 | 5 | 5p15.2 | 14.4 | 16.9 | 2.5 | 6 | rs30617 rs257748 rs250498 rs32826 rs876095 rs890910 | *FLJ11127 LOC391739 EEF1AL11 LOC90268 ANKH LOC402198 LOC391741 FBXL7 LOC441061  LOC401176 ZNF622 FLJ20152 LOC389275 MYO10* |
| 16 | 5 | 5q11.2 | 53.1 | 54 | 0.9 | 5 | rs31226 rs2407511 rs2017318 rs2099082 rs952380 | *ASSP9 ARFRP2 LOC441071 HSPB3 SNAG1 LOC389290* |
| 17 | 6 | 6p25.3 6p24 | 6.3 | 7.4 | 1.1 | 8 | rs1902946 rs554653 rs2182277 rs2182278 rs1886883 rs1750242 rs1324506 rs1324505 | *FLJ33708 LY86 RREB1 SSR1 CTAG3 RIOK1* |
| 18 | 7 | 7p14 | 31.9 | 32.9 | 1 | 8 | rs2392002 rs722271 rs28190 rs28179 rs2190998 rs717757 rs1839574 rs2392147 | *LOC441209 LSM5 KIAA0241 LOC441210 LOC441211 LOC401321 LOC401322  KBTBD2 LOC441212 FKBP9 NT5C3* |
| 19 | 7 | 7p14 | 36.1 | 38.2 | 2.1 | 9 | rs2392383 rs196571 rs4128395 rs2392461 rs2016547 rs1986567 rs1986568 rs718015 rs1376256 | *KIAA0895 ANLN AOAH NPM1P18 ELMO1 LOC442295 GPR141 TXNDC3 SFRP4  UCC1 STARD3NL TRGC2 TRGJ2 TRGJP2 TRGC1 TRGJ1 TRGJP TRGJP1 TRGV11  TRGVB TRGV10 TRGV9 TRGVA TRGV8 TRGV7 TRGV6 TRGV5P TRGV5 TRGV4  TRGV3 TRGV2 TRGV1* |
| 20 | 7 | 7p12 | 47.6 | 49 | 1.4 | 7 | rs2346864 rs2881495 rs2248842 rs953264 rs965694 rs717707 rs1030708 | *PKD1L1 HUS1 MGC33329 LOC136288 UPP1 ABCA13 LOC441223 GDI2P MGC26484* |
| 21 | 7 | 7q11.21 7q11.22 | 66.5 | 68.4 | 1.9 | 5 | rs970761 rs2864461 rs2077872 rs723886 rs2865116 | *LOC441248 LOC441249* |
| 22 | 8 | 8q11.2 | 52.9 | 53.3 | 0.4 | 6 | rs1444161 rs991408 rs1425902 rs1425903 rs2199590 rs1477965 | *LOC115294 LOC389657* |
| 23 | 8 | 8q22 | 100.1 | 101 | 0.6 | 6 | rs1954700 rs719870 rs1954695 rs2088394 rs952296 rs723733 | *LOC441372 COH1* |
| 24 | 8 | 8q24.1 | 119.1 | 121 | 2 | 10 | rs1348724 rs1374098 rs1992721 rs965670 rs2060319 rs1021897 rs1482181 rs1542683 rs1384184 rs1384185 | *SAMD12 LOC441377 TNFRSF11B COLEC10 MAL2 NOV LOC392264 ENPP2 HCP23  TAF2 MGC5528 DEPDC6* |
| 25 | 8 | 8q24.1 | 126.4 | 128 | 1.3 | 16 | rs1826083 rs1565048 rs1914295 rs763319 rs1516951 rs1386468 rs1386469 rs1400458 rs1386470 rs720524 rs958653 rs1378897 rs953668 rs1365371 rs2392827  rs1499364 | *TRIB1 LOC389685 NSE2* |
| 26 | 8 | 8q24.1 | 128.2 | 129 | 0.8 | 6 | rs1073754 rs1356763 rs2395855 rs1074411 rs1030626 rs1368136 | *POU5F1P1 MYC PVT1* |
| 27 | 8 | 8q24.2 | 131.4 | 138 | 6.5 | 17 | rs1392871 rs1878864 rs1868440 rs727489 rs2002265 rs717583 rs1383464 rs2316939 rs1375255 rs961369 rs3911702 rs3844033 rs2610120 rs2582454 rs2256827  rs2256821 rs340708 | *ADCY8 KIAA0143 OC90 HHLA1 KCNQ3 LRRC6 FLJ33069 CGI-72 TG SLA WISP1 NDRG1  LOC392271 FAM10A6 SIAT4A ZNF406 LOC286094 KHDRBS3* |
| 28 | 8 | 8q24.2 | 139.1 | 141 | 1.4 | 5 | rs2111571 rs2319420 rs726348 rs1397381 rs1397380 | *LOC51059 COL22A1* |
| 29 | 9 | 9q31 9q31.3 | 99.8 | 101 | 0.9 | 7 | rs717890 rs1570303 rs1570304 rs1856203 rs1889224 rs1889223 rs1105564 | *TXNDC4 NANOGP5 INVS TEX10 MGC17337 TMEFF1 LOC347273* |
| 30 | 14 | 14q21 14q22 | 49.8 | 50.8 | 1 | 6 | rs1950916 rs726529 rs726531 rs726532 rs1390375 rs1006237 | *ATP5S CDKL1 MAP4K5 SNRPGP SPG3A SAV1 ZNF405P NIN C14orf29 MRP63P9 PYGL  LOC400212 TRIM9 TXNDC* |
| 31 | 14 | 14q22 14q23 | 55.7 | 57.6 | 1.9 | 10 | rs1152537 rs812691 rs1955641 rs178497 rs178138 rs66616 rs1954004 rs721070 rs1951483 rs755011 | *PELI2 LOC400216 C14orf101 OTX2 LOC145414 LOC440180 SEC10L1 C14orf108 C14orf35  C14orf105 SLC35F4 LOC401777 LOC441686* |
| 32 | 17 | 17p13 17p13.2 | 0.9 | 4.3 | 3.4 | 7 | rs1807333 rs1825935 rs1367950 rs1821139 rs966004 rs781852 rs724809 | *ABR MRPL14P1 LOC390754 TUSC5 YWHAE CRK MYO1C SKIP PITPN MGC34680 SCARF1  RILP PRPF8 MGC14376 FLJ33817 SERPINF2 SERPINF1 SMYD4 RPA1 RTN4RL1 DPH2L1  OVCA2 HIC1 C17orf31 LOC440396 SRR FLJ10534 RUTBC1 MNT LOC284009 MGC3329  PAFAH1B1 KIAA0664 GARNL4 OR1D4 OR1D2 OR1E3P OR1G1 OR1P1P OR1A2 OR1A1  OR1D3P OR3A1 LOC390756 OR3A2 OR1R1P OR1E1 OR3A3 OR1E2 NYD-SP20 ASPA  TRPV3 TRPV1 CARKL CTNS TAX1BP3 MGC2963 P2RX5 GSG2 ITGAE HSA277841  CAMKK1 P2RX1 ATP2A3 ZZEF1 MGC32124 ANKFY1 UBE2G1 MGC29671* |
| 33 | 18 | 18p11.31 | 3.2 | 4.4 | 1.2 | 8 | rs727929 rs1380775 rs918174 rs988281 rs976469 rs950959 rs556831 rs1595361 | *LOC440476 MRCL3 MRLC2 LOC440477 LOC204777 TGIF DLGAP1 LOC441807 LOC388458* |
| 34 | 20 | 20p11.1 20q11.1  20q11.23 | 25 | 34.3 | 9.3 | 13 | rs2387577 rs2207631 rs2387733 rs1474945 rs721220 rs725478 rs2378132 rs819144 rs819145 rs725908 rs964786 rs3850528 rs1073768 | *VSX1 LOC391239 LOC284798 ENTPD6 PYGB C20orf22 PPIAP2 KIAA0186 KIAA0980 C20orf147  LOC440760 ZNF337 LOC400840 C20orf189 FLJ38374 LOC149934 LOC284801 FLJ45832 MGC72104  LOC391241 LOC149935 RPL31P3 DEFB118 DEFB119 DEFB123 REM1 LOC388794 HM13 ID1  COX4I2 BCL2L1 TPX2 MYLK2 FKHL18 DUSP15 LOC164395 C20orf126 LOC343702 C20orf160 HCK TM9SF4 RPL24P1 TSPYL3 PLAGL2 POFUT1 KIF3B ASXL1 C20orf112 LOC400842 LOC149950  FLJ33706 BCL2L7P1 COMMD7 DNMT3B MAPRE1 LOC388795 SPAG4L BPIL1 BPIL3 LOC391242  C20orf185 C20orf186 C20orf70 SOCS2P1 BASE C20orf71 PLUNC RPL12P3 C20orf114 C20orf115  CDK5RAP1 LOC440761 SNTA1 CBFA2T2 C20orf144 APBA2BP E2F1 PXMP4 RPL31P2 ZNF341  C20orf178 TPM5P RALY EIF2S2 LOC440762 XPOTP1 ASIP AHCY CDC42P1 ITCH FDXP1 DNCL2A  MAP1LC3A CDC91L1 TP53INP2 NCOA6 HMG4L GGTL3 ACAS2 GSS MYH7B TRPC4AP C20orf31  PROCR MMP24 LOC400843 ITGB4BP C20orf128 C20orf44 GDF5 CEP2 LOC343705 C20orf173  SDBCAG84 FER1L4 RPL37P1 SPAG4 CPNE1 RBM12 NFS1 C20orf52 RNPC2 C20orf53 COX7BP2  C20orf104 SCAND1 C20orf152 HMG4L2 EPB41L1* |
| 35 | 22 | 22q12.1 | 24.9 | 26.1 | 1.2 | 6 | rs734088 rs723184 rs723183 rs1885359 rs720621 rs2078555 | *SEZ6L LOC57168 HPS4 LOC402055 TFIP11 TPST2 CRYBB1 CRYBA4 LOC391325 LOC440823  LOC388889 LOC284898* |
| 36 | 22 | 22q13.1 | 34.5 | 38.6 | 4.1 | 5 | rs763668 rs139897 rs195306 rs926299 rs139480 | *RBM9 RPL41P3 NDUFA9P1 APOL3 APOL4 APOL2 APOL1 MYH9 LOC440828 LOC388898 TXN2  FLJ23322 EIF3S7 CACNG2 RABL4 PVALB FLJ90680 NCF4 CSF2RB CSF2RB2 MGC35206 TST  MPST FLJ12242 TMPRSS6 IL2RB C1QTNF6 SSTR3 RAC2 PSCD4 KIAA1904 MFNG CARD10  CDC42EP1 LGALS2 GGA1 PDXP SH3BP1 LGALS1 MGC3731 HRIHFB2122 H1F0 GCAT GALR3  LOC129138 EIF3S6IP MIRAB13 C22orf23 POLR2F SOX10 PRKCABP SLC16A8 FLJ22582 PLA2G6  MAFF C22orf5 CSNK1E LOC400927 KCNJ4 KDELR3 DDX17 DMC1 LOC388900 C22orf2 TOMM22  KIAA0063 GTPBP1 UNC84B DNAL4 NPTXR CBX6 APOBEC3A APOBEC3B APOBEC3C APOBEC3E  APOBEC3F APOBEC3G ARP10 COX5BL7 CBX7 FLJ23865 PDGFB RNU83B RNU83A RPL3 RNU86  SYNGR1 MAP3K7IP1 MGAT3 FLJ20232 ATF4 MGC52010 CACNA1I FLJ25421* |
